# Two Point Mutations in the Glycoprotein of SFTSV Enhance the Propagation Recombinant Vesicular Stomatitis Virus Vectors at Assembly Step

**DOI:** 10.3390/v15030800

**Published:** 2023-03-21

**Authors:** Qiang Hu, Yuhang Zhang, Jiafu Jiang, Aihua Zheng

**Affiliations:** 1 College of Life Science, Hebei University, Baoding 071002, China; 2 State Key Laboratory of Integrated Management of Pest Insects and Rodents, Institute of Zoology, Chinese Academy of Sciences, Beijing 100101, China; 3 CAS Center for Excellence in Biotic Interactions, University of Chinese Academy of Sciences, Beijing 100101, China; 4 State Key Laboratory of Pathogen and Biosecurity, Beijing Institute of Microbiology and Epidemiology, Beijing 100071, China

**Keywords:** SFTSV, vaccine, vesicular stomatitis virus, mutation, assembly

## Abstract

Severe fever with thrombocytopenia syndrome virus (SFTSV) is an emerging tick-borne pathogen for which approved therapeutic drugs or vaccines are not available. We previously developed a recombinant vesicular stomatitis virus-based vaccine candidate (rVSV-SFTSV) by replacing the original glycoprotein with Gn/Gc from SFTSV, which conferred complete protection in a mouse model. Here, we found that two spontaneous mutations, M749T/C617R, emerged in the Gc glycoprotein during passaging that could significantly increase the titer of rVSV-SFTSV. M749T/C617R enhanced the genetic stability of rVSV-SFTSV, and no further mutations appeared after 10 passages. Using immunofluorescence analysis, we found that M749T/C617R could increase glycoprotein traffic to the plasma membrane, thus facilitating virus assembly. Remarkably, the broad-spectrum immunogenicity of rVSV-SFTSV was not affected by the M749T/C617R mutations. Overall, M749T/C617R could enhance the further development of rVSV-SFTSV into an effective vaccine in the future.

## 1. Introduction

Severe fever with thrombocytopenia syndrome virus (SFTSV) is an emerging zoonotic pathogen first reported in 2009 in China with a fatality rate of 2–30% [1,2,3,4,5,6]. SFTSV is a tick-borne bandavirus originally identified in Dabie Mountain [7,8]. Human cases outside China were confirmed in South Korea, Japan, Vietnam, Myanmar, Thailand, and Pakistan in the subsequent decade [9,10,11,12]. Similar to other bandaviruses, SFTSV is spherical with an envelope and a genome comprising large (L), medium (M), and small (S) fragments, in which the Gn and Gc proteins are encoded by M fragments [8]. SFTSV Gn/Gc glycoprotein is responsible for receptor binding and membrane fusion and is the main target of neutralizing antibodies [13,14,15,16].

SFTSV is mainly transmitted by the *Haemaphysalis longicornis* tick [1,17] but has also been detected in *Amblyomma testudinarium* and *Ixodes nipponensis* in China and Korea [18,19,20,21]. Although the virus is predominantly transmitted by tick bites, human–human transmission through close contact has been reported [22]. Ferret model studies have revealed that SFTSV can be transmitted by body fluids, such as urine and saliva [23]. Recently, we found that hedgehogs are the major amplifying hosts [24]. The symptoms of SFTSV infection include fever, muscle pain, vomiting, diarrhea, and thrombocytopenia [1,25]. No licensed vaccines and anti-viral medicines exist that prevent SFTSV infection.

Many SFTSV vaccines are under pre-clinical development, such as subunit, attenuated, viral vector, and DNA vaccines; protective effects have also been verified in various animal models. A highly attenuated vaccinia virus strain LC16m8-based vaccine can protect immunodeficient interferon α/β receptor knockout (IFNAR^−/−^) mice against challenge with 1 × 10^5^ tissue culture infectious dose (TCID50; 50% tissue culture infectious dose) of the SFTSV-YG1 strain [26]. In 2020, Korean researchers reported that immunization with single-plasmid DNA encoding IL-12 and SFTSV antigens conferred complete protection in IFNAR^−/−^ mice [27]. A bivalent recombinant human adenovirus type 5-based vaccine expressing rabies virus G and SFTSV Gn elicited protective immunity in mice against rabies virus and SFTSV [28]. Young Ki Choi et al. from South Korea reported a lethal infection ferret model and found an SFTSV DNA vaccine could provide complete protection against SFTSV in fatally infected ferrets [29,30]. Further, using this ferret model, they confirmed the protective efficacy of two live attenuated vaccines [31]. However, none of these vaccine candidates were approved for clinical trials. Regarding anti-viral inhibitors, Zhiwei Wu et al. from Nanjing University developed a camel anti-SFTSV single-domain neutralizing antibody that can block SFTSV in vivo and alleviate virus-induced thrombocytopenia [32].

VSV is a rhabdovirus, which is an enveloped, negative-sense, single-stranded RNA virus approximately 11 kb in size. The VSV genome encodes five structural proteins, nucleocapsid protein (N), phosphoprotein (P), matrix protein (M), glycoprotein (G), and large polymerase protein (L). VSV causes mild disease, such as vesicular lesions around the mouth, hooves, and teats of livestock, including horses, cattle, and pigs [33,34]. VSV is a promising vaccine vector due to the characteristics of high immunogenicity, the lack of pre-existing population immunity, a small genome, and easy manipulation and production scale-up [35,36,37]. VSV-ZEBOV (Ervebo) was the first viral-vector vaccine approved by the Food and Drug Administration of the USA in 2019 for the control of the Ebola virus [38]. More VSV vector vaccines, such as VSV-Indiana HIV, rVSV∆G-LASV-GPC, rVSV-Nipah, and IIBR against SARS-CoV-2, are being evaluated in clinical trials (NCT01438606, NCT04794218, NCT05178901, and NCT04990466, respectively).

**Figure 1:**
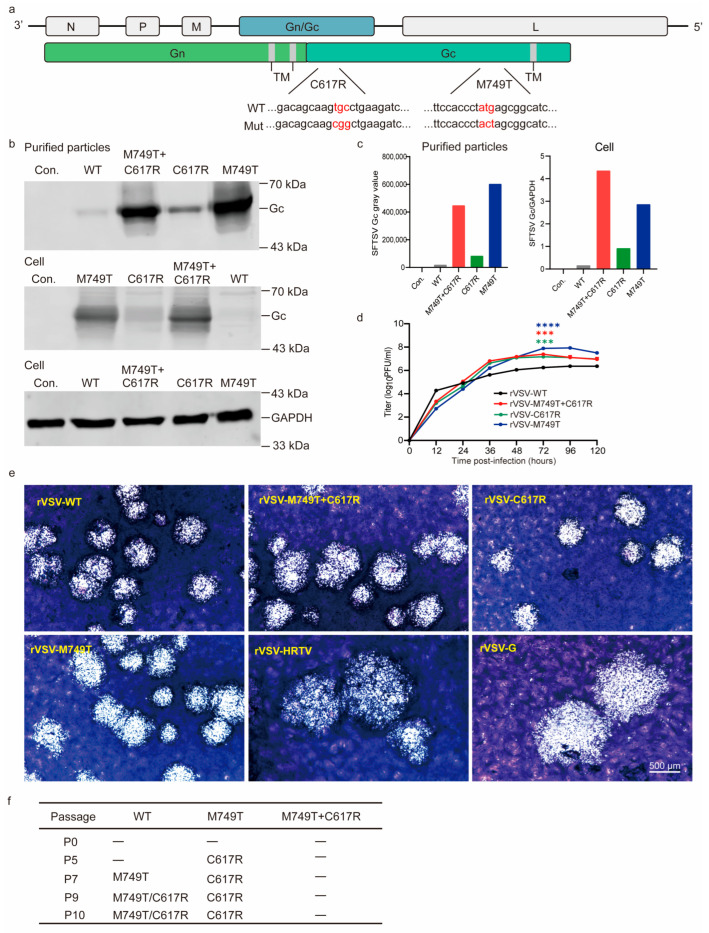
Characterization of rVSV-SFTSV variants. (**a**) Schematic of the mutations that emerged in the SFTSV Gc protein. Mutation (Mut). Mutated nucleotides (Red) (**b**) Gc expression in the supernatants and cell lysates of rVSV-SFTSV WT- and variant-infected Vero cells (MOI = 0.01). Forty-eight hours post-infection, purified particles and cell lysates were blotted with anti-SFTSV Gc polyclonal antibodies (GAPDH as control). Con. indicates mock-infected cells. (**c**) Quantification of (**b**). (**d**) Growth kinetics of rVSVs (MOI = 0.01). *** *t* test, *p* < 0.001; **** *t* test, *p* < 0.0001. (**e**) Representative image of plaques formed using rVSV-SFTSV variants (6 days), rVSV-HRTV (6 days), and rVSV-G (3 days) at indicated days post-infection in Vero cells. Scale bar: 500 μm. (**f**) Genetic stability of rVSV-WT and rVSV-M749T + C617R during passages (P) in Vero cells. The above data are representative of three independent experiments.

In our previous study, we developed a VSV-vector SFTSV vaccine by replacing the VSV glycoprotein (G) gene with the SFTSV Gn/Gc gene and named it rVSV-SFTSV/AH12-GP (Figure 1a). This vaccine was highly attenuated and could induce strong and broad-spectrum protective immunity against SFTSV and Heartland virus (HRTV) [39]. However, rVSV-SFTSV/AH12-GP only propagated to 3 × 10^6^ PFU/mL in Vero cells, limiting its further development. Here, we identified two mutations in the Gc protein that significantly enhance the titer to 7 × 10^7^ PFU/mL without affecting the immunogenicity.

## 2. Materials and Methods

### 2.1. Viruses, Antibodies, and Cells

SFTSV Wuhan strain (GenBank accession numbers: S, KU361341.1; M, KU361342.1; and L, KU361343.1) and rabbit anti-SFTSV-NP polyclonal antibodies were gifts from Dr. Fei Deng in Wuhan Institute of Virology, Chinese Academy of Sciences. Anti-SFTSV Gc polyclonal antibodies were gifts from Dr. George F. Gao and Dr. Yan Wu, Institute of Microbiology, Chinese Academy of Sciences. Vero cells were obtained from Shenzhen Kangtai Biological Products Co., Ltd. (Shenzhen, China) and maintained in Dulbecco’s modified Eagle’s medium (DMEM, Hyclone) supplemented with 8% FBS at 37 °C with 5% CO_2_. 293T cells were obtained from ATCC (CRL-3216) and maintained in Dulbecco’s modified Eagle’s medium (DMEM, Hyclone) supplemented with 8% FBS at 37 °C with 5% CO_2_

### 2.2. Construction and Rescue of rVSVs

The rVSVs were constructed and rescued as previously reported [40,41,42]. rVSV-M749T + C617R, rVSV-M749T, and rVSV-C617R mutant plasmids were constructed using primers converting cysteine at the Gn/Gc 617 locus to arginine (TGC ⟶ CGG) and methionine at the 749 locus to threonine (ATG ⟶ ACT). Here, 2  ×  10^6^ 293T cells were cultured in 6 cm dish one day before transfection and transfected at about 80% confluence. Viruses were rescued by transfecting 1.6 µg rVSV plasmid and five helper plasmids T7 (8.1 μg), N (1.286 μg), P (639 ng), M (169.9 ng), and L (169.9 ng) into 293T cells using calcium phosphate precipitation. Fresh DMEM medium (containing 2% fetal bovine serum) was replaced at 24 h post-transfection. Viral RNAs were extracted from the supernatants and viruses were verified using sequencing at each passage.

To purify the rVSV viral particles, supernatants containing rVSVs were collected 48 h post-transfection and loaded into a 12.5 mL ultra-centrifugation tube on a 15% sucrose cushion. After centrifugation at 30,000 rpm for 3 h at 4 °C (Beckman SW-40 rotor, Brea, CA, USA), the pellets were resuspended in PBS to recover the virus. The cells were lysed on ice with RIPA buffer (10 mM Tris-Cl (pH 8.0), 1 mM EDTA, 0.5 mM EGTA, 1% Triton X-100, 0.1% sodium deoxycholate, 0.1% SDS, 140 mM NaCl, and 1 mM PMSF) for 10 min and centrifuged for 10 min at 4 °C. Samples were run on 10% SDS-PAGE and transferred to the nitrocellulose filter membrane. Anti-SFTSV Gc polyclonal antibodies were applied to detect Gc expression in purified virus particles and cell lysates. Anti-GAPDH antibodies were used as input control.

### 2.3. Growth Kinetic of rVSVs

Vero cells were seeded into T75 flasks at a density of 3 × 10^6^ cells per flask 24 h before infection and then infected with rVSVs at an MOI of 0.01. After 3 h, the culture medium was replaced with fresh DMEM (containing 2% fetal bovine serum) and cultured at 28 °C. Supernatant samples were collected every 12 h and stored in a −80 °C freezer, and the titers were measured using a plaque assay at the same time.

### 2.4. Plaque Assay

Vero cells were seeded into 24-well plates at a density of 100,000 cells per well 24 h before infection. Virus samples were 10-fold diluted with DMEM plus 2% FBS and loaded into 24-well plates for 3 h in a 37 °C incubator. Then, the medium was replaced with 1.3% methylcellulose medium and incubated at 28 °C. Six days later, cells were fixed with 4% paraformaldehyde for 10 min and stained with crystal violet.

### 2.5. Immunofluorescence Microscopy

The DsRed-KDEL plasmid encoding DsRed fused with the ER targeting sequence of calreticulin at the N-terminus and an ER retention sequence (KDEL) at the C-terminus was transfected into Vero cells using Fugene6 (Promega, Madison, WI, USA). Twenty-four hours later, the transfected cells were infected with rVSV-WT, rVSV-M749T + C617R, rVSV-M749T, and rVSV-C617R mutants, respectively. The cells were then fixed, permeabilized, and stained with rabbit anti-SFTSV-Gc polyclonal antibodies (1:1000 dilution in PBS with 3% BSA).

Vero cells were seeded into chamber slides and infected with rVSV-WT and variants within 24 h. Sixteen hours after infection, the plates were cooled on ice for 10 min and blocked with DMEM plus 10% FBS for 30 min on ice. Live Vero cells were incubated with anti-SFTSV-Gc polyclonal antibodies for 1 h and stained with anti-rabbit Alexa Fluor^®^ 488 fluorescent antibody (Invitrogen, Waltham, MA, USA) at 4 °C. Cells were fixed with 4% paraformaldehyde, and nuclei were stained with Hoechst 33342 (Invitrogen). Images were obtained with a LEICA Stellars 5 laser scanning confocal microscope.

### 2.6. Animal Experiments and Immunization

C57/BL6 IFNAR^−/−^ mice were purchased from the Institute of Laboratory Animals of the Chinese Academy of Medical Sciences. The male and female mice were randomly divided into three groups (*n* = 5) and i.p. immunized at two points with a dose of 2 × 10^4^ PFU. Control groups were mock immunized with a DMEM medium. Body weights and clinical symptoms were monitored for 7 days after immunization. Sera samples were taken 28 days after immunization for the neutralizing assay. The immunized mice were challenged with the SFTSV Wuhan strain at a dose of 2 × 10^4^ FFU on day 30. Whole blood samples were collected for viremia analyses on 1, 3, and 5 days post-challenge.

### 2.7. Neutralizing Assay

Inactivated sera samples were 3-fold serially diluted and incubated with 150 FFU of rVSVs with a GFP reporter for 30 min by duplicates. The mixtures were then added to a 96-well plate containing Vero cells, and the plate was incubated at 37 °C for 3 h. After one wash, the cells were covered with a 1.3% methylcellulose medium overlay at 28 °C. GFP-positive cells were counted 20 h post-infection using the Opera Phenix High Content Screening System (PerkinElmer, Waltham, MA, USA), and NAb titers were calculated as FRNT50 using the Reed–Muench method.

### 2.8. Real-Time PCR

Total RNA was extracted from whole blood samples uing the TIANamp Virus RNA Kit (Tiangen) following the manufacturer’s instructions. RNA samples were analyzed using a One Step SYBR PrimerScript reverse transcription (RT)-PCR kit (TaKaRa) on Applied Biosystems QuantStudio. The β-actin gene was used as the reference, and the relative expression level was calculated using the standard ΔΔ-CT method. β-Actin primer details are as follows: forward, GGCTGTATTCCCCTCCATCG; reverse, CCAGTTGGTAACAATGCCATGT. SFTSV Wuhan strain primer details are as follows: forward, ATGGATAGCAGCGTCTCATCAAATC; reverse, TGAGCGCACTGTATGAGGTAGGTAA. Real-time quantitative PCR was initiated at 42 °C for 5 min and incubated at 95 °C for 10 s, followed by 40 cycles at 5 s at 95 °C, and 20 s at 60 °C.

## 3. Results

### 3.1. Two Point Mutations in the Gc Glycoprotein Enhance the Titer rVSV-WT

Previously, we found that rVSV-SFTSV/AH12-GP is genetically stable in Vero cells across six passages with a peak titer of 3 × 10^6^ PFU/mL. However, the viral titer reached 8 × 10^7^ PFU/mL when we continued to passage seven. Sequencing analyses revealed that an M749T substitution emerged in the Gc protein. In addition, another substitution in Gc, C617R, was found at passage nine with no significant increase in virus titer.

To investigate the impact of the two mutations on virus propagation, we introduced single or double mutations into rVSV-SFTSV/AH12-GP (assigned rVSV-WT, rVSV- C617R, rVSV-M749T, and rVSV-M749T + C617R). All the mutants were successfully rescued in Vero cells. Forty-eight hours after the infection of Vero cells with rVSV-WT and mutants, SFTSV Gc expression in the cell lysates and particles purified from the supernatants was confirmed with western blotting using polyclonal antibodies. As shown in Figure 1b,c, Gc expression was significantly increased using the M749T mutation and further slightly enhanced using the C617R mutation. To assess the impact of the mutations on virus propagation, we obtained the growth kinetics of rVSV and the mutants in Vero cells (Figure 1d). Within 24 h after infection, the titers of mutants were no higher than that of rVSV-WT; however, after 24 h, the titers of the three variants were consistently higher than that of the WT strain, and the rVSV-M749T titer was highest, peaking at 8 × 10^7^ PFU/mL. The peak titer of rVSV-C617R was slightly lower than that of rVSV-M749T but close to that of rVSV-M749T + C617R. As shown in Figure 1e, the rVSV-WT and mutants formed similar plaque sizes in Vero cells but were smaller than those formed using rVSV-HRTV and rVSV-G. rVSV-M749T + C617R was genetically stable, and no further mutations appeared in the Gn/Gc protein after 10 passages, suggesting that the mutant viruses had adapted to Vero cells (Figure 1f).

Although rVSV-M749T showed the highest titer, this construct is genetically unstable, and the C617R mutation always emerges within five passages. In contrast, rVSV-M749T + C617R was stable after 10 consecutive passages. Thus, we hypothesize that rVSV-M749T + C617R is a promising candidate for vaccine development.

### 3.2. The M749T and C617R Mutations Promote rVSV-SFTSV Assembly by Increasing Glycoprotein Localization at the Plasma Membrane

Assembly is a key bottleneck for the development of rVSV-vector recombinant virus when the assembly occurs in different cellular compartments. VSV assembles and buds from the plasma membrane, while SFTSV buds from the membrane of the Golgi complex, similar to other Bunyaviruses [43,44,45]. In a previous study, two mutations in the glycoprotein of Hantavirus, a Bunyavirus, relocated Gn/Gc from the Golgi complex to the cell surface and, in turn, enhanced Gn/Gc incorporation into budding VSV particles [46]. We evaluated the localization of Gn/Gc proteins within cells to explore how the mutations M749T and C617R enhance the titer of rVSV-SFTSV.

**Figure 2:**
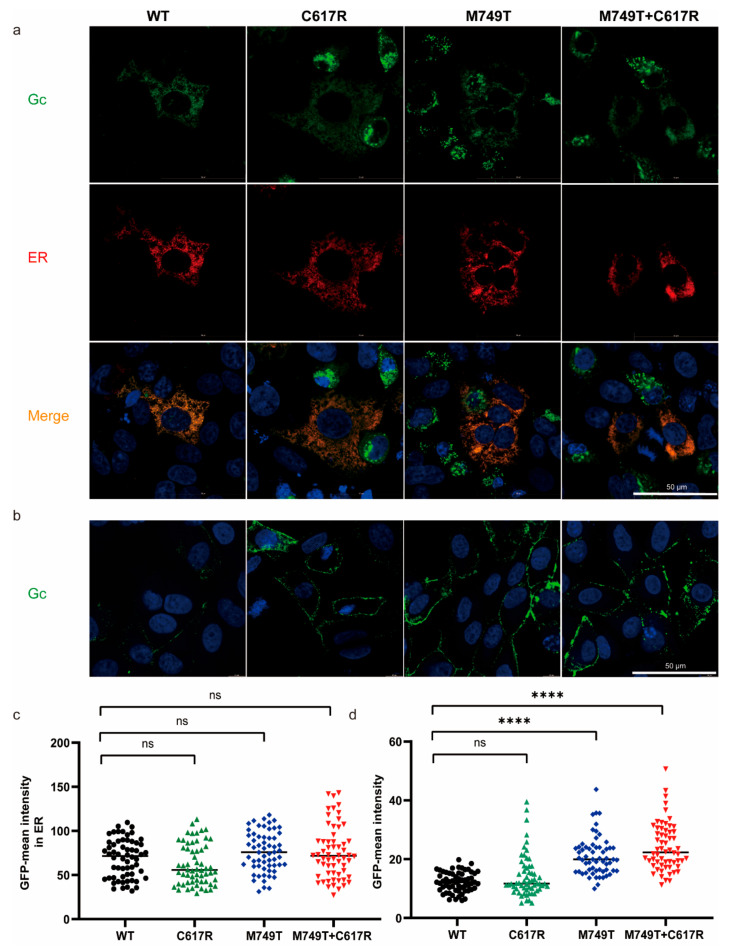
The effect of M749T and C617R mutations on the plasma membrane localization of Gc. (**a**) Vero cells were infected with rVSV-WT and variants 24 h after transfection with a plasmid encoding the ER reporter DsRed-KDEL (red) and stained with anti-Gc polyclonal antibodies (green) 24 h post-infection. (**b**) Live Vero cells were infected with rVSVs for 16 h and stained with anti-Gc polyclonal antibodies (green). (**c**) Quantification of the density of Gc in the whole cell from (**a**) (*n* = 60). (**d**) Quantification of Gc in plasma membrane from (**b**) (*n* = 60). Statistical significance was determined using an unpaired Student’s *t*-test. ns, *p* > 0.05; **** *p* < 0.0001. The above data are representative of three independent experiments.

To determine the localization of mutant SFTSV glycoprotein, we transiently expressed the DsRed–KDEL fusion protein in Vero cells. The red fluorescent protein was targeted to the endoplasmic reticulum (ER) by N-terminal fusion to the signal peptide and retained there via a C-terminal KDEL-tag [47]. The cells were infected with rVSV-WT, rVSV-M749T, rVSV-C617R, or rVSV-M749T + C617R 24 h after transfection. As shown in Figure 1d, the titers of the mutant virus were no higher than those of the WT; thus, we analyzed the Gc protein localization within 24 h post-infection. Twenty-four hours post-infection, the Vero cells were fixed and stained using an antibody against Gc protein, while the nuclei were stained with Hoechst33342. Most of the mutant glycoproteins were localized in the ER, similar to the situation with the WT virus (Figure 2a). The quantitative analysis revealed no significant differences between the expression levels of WT and mutant Gc proteins (Figure 2c). Thus, M749T and C617R do not play a role in protein expression.

We further assessed the effect of M749T and C617R on the membrane localization of the Gc protein. Live Vero cells were stained with anti-Gc polyclonal antibodies 16 h post-infection with WT or mutant viruses. We found that M749T significantly enhanced the level of Gc protein on the plasma membrane, while no obvious difference was observed with C617R. Additionally, there was no difference between rVSV-M749T and rVSV-M749T + C617R. However, we found the Gc protein expression in rVSV-C617R showed higher variation than rVSV-WT (Figure 2b,d). Overall, these data demonstrate that M749T + C617R enhanced the titer of rVSV-SFTSV by facilitating the membrane trafficking of glycoproteins without affecting the protein expression levels.

### 3.3. M749T and C617R Do Not Affect the Immunogenicity of rVSV-SFTSV in Mice

**Figure 3:**
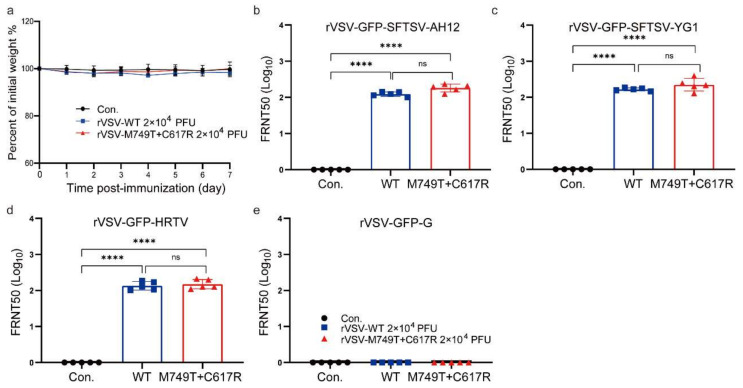
The humoral responses in mice elicited using rVSV-WT and rVSV-M749T + C617R. Three groups of C57/BL6 IFNAR^−/−^ mice (*n* = 5 per group) were i.p. immunized with rVSV-WT, rVSV-M749T + C617R, and DMEM (2 × 10^4^ PFU). (**a**) Mouse body weight changes after immunization. (**b**) NAb titers were determined against rVSV-GFP-SFTSV AH12 (**b**), rVSV-GFP-SFTSV YG1 (**c**), rVSV-GFP-HRTV (**d**), and rVSV-GFP-G (**e**) 28 days after immunization. Titers were calculated using the Reed–Muench method. The above data are representative of two independent experiments. The *p*-value was determined using a two-sided multiple *t*-test; **** *t*-test, *p* < 0.0001.

Mutated glycoproteins can potentially decrease immunogenicity. Groups of IFNAR^−/−^ C57/BL6 mice were intraperitoneally (i.p.) immunized with a single dose of 2 × 10^4^ PFU rVSV-WT, rVSV-M749T + C617R, or a medium control to evaluate the effect of M749T and C617R on the immunogenicity of the rVSV-SFTSV. Body weight was monitored for 1 week, and no obvious weight loss or other clinical signs were observed (Figure 3a).

Blood samples were obtained 28 days post-immunization and neutralizing activities were measured with a 50% focus reduction neutralization test (FRNT50). Our previous study showed that rVSV-WT can elicit a strong and broad-spectrum humoral immune response against the SFTSV Chinese clade strain AH12, the SFTSV Japanese clade strain YG1, and HRTV in the same mouse model [39]. Here, we applied rVSV-GFP-SFTSV, rVSV-GFP-SFTSV-YG1, rVSV-GFP-HRTV, and rVSV-GFP-G for the FRNT assay. Our results demonstrate that rVSV-M749T + C617R elicits a neutralizing titer comparable to that of rVSV-WT against all the viruses tested (Figure 3b–e). Thus, M749T + C617R mutations did not impede the immunogenicity of rVSV-SFTSV.

### 3.4. rVSV- M749T + C617R Protects IFNAR^−/−^ Mice from Lethal SFTSV Infection

**Figure 4:**
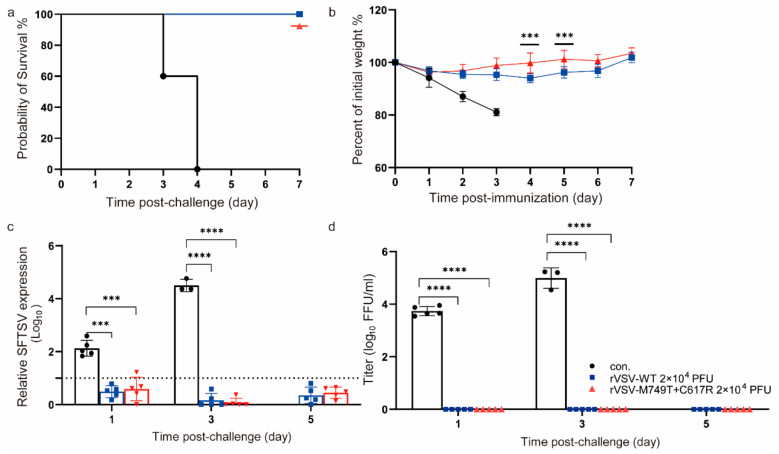
rVSV-WT and rVSV-M749T + C617R confer complete protection against lethal SFTSV Wuhan strain challenge in mice. (**a**) Survival rate of the mice after challenge. Immunized mice were i.p. challenged with a lethal dose of SFTSV (2 × 10^4^ PFU) 30 days after immunization. (**b**) Body weight changes in mice after SFTSV challenge. Data are presented as the means  ±  SD. (**c**) SFTSV viral RNAs in the mice after challenge as measured using real-time PCR. The black bar is the mock-immunized control (con.); The dotted line represents the detection limit. (**d**) Viremia in the sera was measured using a plaque assay in Vero cells. Statistical significance was determined using multiple *t*-test. The above data are representative of two independent experiments. The *p*-value was determined using a two-sided multiple *t*-test; **** *t*-test, *p* < 0.0001; *** *t*-test, *p* < 0.001.

The vaccinated animals were challenged with a lethal dose of 2 × 10^4^ FFU (higher than 10^3^ median lethal dose) SFTSV Wuhan strain via the i.p. route 30 days post-immunization and observed for the development of clinical signs and weight loss. All the mice vaccinated with rVSV-WT or rVSV-M749T + C617R survived, whereas all the control mice died within 3 days post-infection (Figure 4a). All the mice inoculated with either rVSV-WT or rVSV-M749T + C617R developed no obvious clinical signs during the 7-day monitoring period after the SFTSV challenge, while all the control mice showed clinical signs, such as hunched posture, an inability to stand, and rapid weight loss (Figure 4b). SFTSV viremia was determined using reverse transcription (RT)-qPCR at days 1, 3, and 5 post-challenge. Viremia increased quickly from day 1 to day 3 until the mice in the control group died, while no viremia was detected in the vaccinated animals. Infectious viral particles were also detected in the control group by plaque and showed the same trend as determined using PCR. Thus, both rVSV-WT and rVSV-M749T + C617R elicited sterilizing immunity in the mice (Figure 4c,d).

## 4. Discussion

VSV is a promising vaccine vector, especially for highly pathogenic viruses. By replacing the original G glycoprotein with those of the target viruses, the recombinant VSV virus displays the heterologous glycoprotein on the surface, which can trigger a robust immune response. Many virus groups are successfully recombined with VSV, including filoviruses, bunyaviruses, paramyxoviruses, and arenaviruses [38,46,48,49]. However, the titers of many recombinant VSV-vector viruses are not high enough for large-scale production, which is critical for vaccine development.

As previously reported, rVSVs bearing Gn/Gc from the old world hantaviruses, such as the Hantaan virus (HTNV) or the Dobrava-Belgrade virus, were refractory to rescue. Similar to that observed with rVSV-SFTSV, two point mutations in the glycoproteins emerged during serial passage of the rescued rVSV-HTNV virus and markedly increased its infectivity. Of the two mutations, I532K is located in the intracellular domain of Gn and S1094L in the membrane-proximal region of Gc. Further mechanistic studies revealed that these two mutations can re-localize HTNV Gn/Gc from the Golgi complex to the plasma membrane, thereby significantly enhancing Gn/Gc incorporation into budding VSV particles [46]. In our study, both M749T and C617R are located in the Gc protein. M749T can significantly enhance the titer of rVSV-SFTSV, while only a marginal increase is observed with C617R. However, the C617R mutation makes rVSV-M749T + C617R genetically stable in Vero cells, which is important for vaccine development.

RNA viruses have high-mutation rates due to their lack of proofreading activity during replication [50,51]. These mutations can potentially alter immunogenicity [50,52]. For example, the Omicron strain of severe acute respiratory syndrome coronavirus 2 (SARS-CoV-2) is continuously evolving, leading to many new subvariants demonstrating strong neutralizing escape from existing immune responses established by infections and vaccines [53,54]. To explore whether the mutations in the Gc protein affect the immunogenicity of rVSV-SFTSV, we compared the efficacy of rVSV-WT and rVSV-M749T + C617R in IFNAR^−/−^ mice, an established animal model for SFTSV. Fortunately, both rVSV-WT and rVSV-M749T + C617R elicited sterile protection against lethal challenge, with comparable broad-spectrum neutralizing activity against SFTSV of Chinese and Japanese lineages and HRTV. As previous reported, rVSV-SFTSV also induces strong cellular immunity [39], which is more tolerated to mutations than humoral immunity. In the case of SARS-CoV-2, Omicron accumulated large amount of mutations, which significantly affected its humoral immunity. However, the cellular immunity induced by Omicron variant is similar to those of prototype, Beta and Delta [55]. Therefore, we assume that there is no significant difference in the cellular immunity of rVSV-SFTSV between rVSV-M749T + C617R and the wildtype. In summary, M749T + C617R mutations can significantly increase the titer and genetic stability of rVSV-SFTSV, without affecting its immunogenicity, which is beneficial for future vaccine development.

## Data Availability

The published article includes all datasets generated or analyzed during this study.
